# miR-29c inhibits metastasis of gastric cancer cells by targeting VEGFA

**DOI:** 10.7150/jca.77727

**Published:** 2022-10-31

**Authors:** Beiqin Yu, Nan Zhu, Zhiyuan Fan, Jianfang Li, Yani Kang, Bingya Liu

**Affiliations:** 1 Department of General Surgery, Shanghai Key Laboratory of Gastric Neoplasms, Shanghai Institute of Digestive Surgery, Ruijin Hospital, Shanghai Jiao Tong University School of Medicine, Shanghai 200025, China.; 2 School of Biomedical Engineering, Bio-ID Center, Shanghai Jiao Tong University, Shanghai 200240, China.

**Keywords:** gastric cancer, miR-29c, VEGFA, epithelial-mesenchymal transition, cancer stem cells

## Abstract

**Background:** Gastric cancer (GC) is characterized by tissue invasion and metastasis, which lead to an aggressive and highly lethal disease. However, the underlying molecular mechanism remains largely unclear. Although multiple miRNAs are known to regulate crucial cellular events during cancer metastasis, their individual roles are still not fully described.

**Methods:** miR-29c overexpressed cell lines were constructed. The wound healing, migration and invasion assays were performed to investigate the effect of miR-29c on metastasis of GC. HUVECs proliferation and tube formation assays were used to test the ability of angiogenesis of miR-29c. The target gene VEGFA was predicted by bioinformatic algorithms and validated by luciferase activity assay. Peritoneal spreading and pulmonary metastasis mice models were applied *in vivo*.

**Results:** In the current study, we report the results that introduction of exogenous miR-29c inhibits GC cell migration, invasion and angiogenesis. Epithelial-mesenchymal transition (EMT) and cancer stem cells (CSCs) properties are participated in the miR-29c mediated cell metastasis. Furthermore, by performing tumor metastasis PCR array and luciferase reporter assay, we find that the expression of VEGFA is regulated by miR-29c through direct targeting of its 3'-UTR. In addition, we show that the VEGFA/VEGFR2/ERK pathway is involved in this process.

**Conclusion:** These data taken together reveal the crucial functions of miR-29c-VEGFA/VEGFR2/ERK signaling axis in the metastasis progression of GC via regulating EMT and CSCs properties, which make them potential targets for clinical intervention in GC.

## Introduction

Gastric cancer (GC) is one of the most common digestive system malignancies associated with high incidence and mortality rate 1. While great achievements have made major advances in screening, most GC patients were diagnosed with unresectable, locally advanced or metastatic disease and remained to have a poor prognosis 2, 3. The metastasis of GC is the main cause of death for patients 4. Thus, it is of great significance to improve the understanding of the mechanism of metastasis in the prevention and treatment of GC.

MicroRNAs (miRNAs) are short (~18-25 nucleotides) single stranded non-coding RNA molecules, which post-transcriptionally regulate the expression of genes by inhibiting protein translation from mRNA or promoting degradation of mRNA 5. Accumulating evidence demonstrates that miRNAs can modulate multiple biological processes, play important roles in tumor development and metastasis 6, 7. Many efforts have been devoted into the investigation of miRNAs, especially in the area of metastasis 8; however, only limited information about the metastatic property of miR-29 family in GC has been provided. Although miR-29c has been found to be downregulated in GC tissues and inhibited cancer cell proliferation 9, its function in metastasis is still not clarified. Therefore, the role of miR-29c in metastasis deserves further investigation.

Vascular endothelial growth factor (VEGF) and its receptors are essential to tumor angiogenesis and neovascularization, which in turn contributes to the generation and development of cancer metastasis 10, 11. VEGFA, the most functional isoform of VEGF family, exerts its effects by cooperating with VEGFR2 to drive tumor development 12. VEGFA is overexpressed in a variety of tumors and its higher expression correlates with poor prognosis and death from metastasis.

In this study, we intend to investigate the function of miR-29c in GC metastasis and if miR-29c directly targets VEGFA to regulate GC cancer cell metastasis. Our results show that miR-29c inhibits gastric cancer cell migration, invasion, angiogenesis and cancer stem cells (CSCs) properties. Using tumor metastasis PCR array and luciferase reporter assay, we find that miR-29c directly targets the 3'-UTR of VEGFA and plays critical roles in the process of metastasis inhibition through VEGFA/VEGFR2/ERK pathway.

## Materials and Methods

### Cell culture

Human gastric cancer cell lines SGC7901 and MKN28, human umbilical vein endothelial cells (HUVECs) were obtained from Shanghai Institutes for Biological Sciences, Chinese Academy of Sciences. Human embryonic kidney 293T cells (HEK-293T) were purchased from the American Type Culture Collection (ATCC; Manassas, VA, USA) and stored at our lab. SGC7901 and MKN28 cells were cultured in RPMI-1640 medium, while HEK-293T and HUVEC cells were maintained in DMEM medium, supplemented with 10% fetal calf serum (FBS; Invitrogen, Carlsbad, CA, USA), 100 U/ml penicillin and 100 µg/ml streptomycin in a humidified environment containing 5% CO2 at 37 °C.

### Plasmids and lentiviral infection

Cells were infected with lentivirus harboring plasmids of miR-29c or miR-control, VEGFA or vector, to generate an ectopic expression of miR-29c and VEGFA and their respective control groups. Lentiviral package and infection procedure was carried out as described before 9.

### Wound healing assay

Cells transfected with miR-29c or VEGFA (and respective controls) were seeded on 6-well plates. The confluent monolayer was scratched with a pipette tip to generate a wound and then incubated in fresh RPMI-1640 medium to induce migration. Pictures of wounds were taken every 24 h and wound closure was measured by Image J.

### Cell migration and invasion assay

Transwell chambers (Corning, Lowell, MA, USA; 8 μm, 24-well insert) were used to perform cell migration and invasion assays. Stably transfected cell lines were incubated for 24 h in serum-free medium, then 2 × 10^4^ cells were suspended in 200 μl serum-free medium and seeded in the upper insert chamber within 600 μl medium containing 10% FBS in the lower chamber. Diluted Matrigel (BD Biosciences, Franklin Lakes, NJ, USA) were coated on the insert membranes for invasion assay. After incubation at 37 °C, 5% CO2, non-migrated/invaded cells were removed with cotton-tipped swabs and cells that migrated/invaded to the bottom of the membranes were fixed in 4% paraformaldehyde and stained with 0.5% crystal violet solution. After staining, the cells were photographed at least five randomly selected fields with an inverted microscope and counted with Image J.

### Cell proliferation assay

HUVECs were seeded in 96-well tissue culture plates overnight and then were treated with tumor-conditioned medium. Proliferation was measured using the CCK8 assay (Dojindo Laboratories, Japan) according to the manufacturer's instructions. The absorbance was measured at a wavelength of 450 nm.

### Endothelial tube formation assay

Assays were performed using the endothelial tube formation kit (Cell Biolabs, San Diego, CA, USA) to assess angiogenesis *in vitro*. A prechilled 96-well tissue culture plate was coated with a thin ECM gel layer (50 µl/well), and then polymerized at 37°C for 1 h. HUVECs (2 × 10^4^ cells for each well) that resuspended in collected supernatants from SGC7901-miR-29c or SGC7901-miR-control were added to the prepared gel with 300 µl supernatants. Tubules were visualized under microscopy and evaluated by Image J after 12 h incubation at 37 °C with 5% CO2.

### Immunohistochemistry

Blocks of formalin-fixed, paraffin-embedded mouse subcutaneous tumors were used. Tissue sections (5 μm) were deparaffinized with xylene, rehydrated in ethanol, antigen retrieval was performed by boiling in 10 mM citrate buffer (pH 6.0) for 30 min. After inhibition endogenous peroxidase activity with 0.3% H_2_O_2_ for 10 min, sections were blocked in 2% serum in PBS for 30 min, incubated with monoclonal rat anti-mouse CD31 (1:50; BD Bioscience) at 4 °C overnight, followed by secondary antibody incubation and visualized with Envision System Dako, Carpinteria, CA, USA). Sections were counterstained with hematoxylin. All sections were observed and imaged with a microscope (Carl Zeiss, Germany).

### Metastasis assay *in vivo*

Two patterns of metastasis were observed in this experiment, including peritoneal spreading and pulmonary metastasis. Male BALB/c nude mice (4 - 5 weeks old, purchased from the Institute of Zoology, Chinese Academy of Sciences, Shanghai, China) were housed at SPF environment in the Animal Experimental Center, Ruijin Hospital, Shanghai Jiao Tong University School of Medicine. All animal research study and experimental protocol were performed in accordance with animal research principles and institution's guidelines. In both studies, 10 mice were used in each group. SGC7901-miR-29c or SGC7901-miR-control cells (2×10^6^/100 µl PBS) were injected intraperitoneally or into caudal vena. Mice were euthanized by cervical dislocation on the 30th day after intraperitoneal injection or on the 60th day after caudal vena injection. The abdominal masses and the pulmonary metastatic masses were examined carefully.

### Quantitative real-time PCR (qRT-PCR) and tumor metastasis PCR array

Total RNA was extracted with Trizol reagent (Invitrogen), reverse transcription was performed with 1 μg RNA using the SuperScript II reverse transcriptase (Invitrogen) and qRT-PCR was determined by SYBR Green Mastermix (Qiagen, Venlo, Netherlands). Primer sequences are available in Table S1. For tumor metastasis PCR array, the RT products were used as templates for the tumor metastatic factor profiling PCR amplification using the kit from SuperArray Bioscience (PAHS-028, Frederick, MD).

### Western blot

Total protein was extracted with RIPA buffer (Solarbio, Beijing, China). Western blot was carried out by standard protocol with primary antibodies against VEGFA (1:1000, Abcam), ERK1/2 (1:1000, CST), p-ERK1/2 (1:1000, CST), VEGFR2 (1:1000, CST) and p-VEGFR2 (1:1000, CST). Goat anti-mouse/rabbit secondary antibody coupled to horse radish peroxidase (HRP, ProteinTech Group, USA) dilutions were 1:10,000. Primary antibody against β-actin was used as the endogenous control to confirm equal protein loading.

### Sphere formation

Cells were suspended sparsely and plated at a density of 2000 cells/well in 6-well ultralow attachment plates (Corning). The sphere culture medium was composed of Neural Basal medium supplement with B-27, EGF (20 ng/ml), bFGF (10 ng/ml) and heparin (5 μg/ml). The medium changed every other day and the spheres were observed and counted on an inverted microscope. Sphere formation efficiency was calculated as a percentage of the number of spheres formed from 2000 cells.

### Luciferase activity assay

The VEGFA-3'UTR sequences were synthesized by Sangon (Shanghai, China) with the corresponding miR-29c binding site either left as wild-type sequences (WT) or mutated to abolish miR-29c binding (MUT). Wild type and mutant VEGFA-3'UTR were digested by MluI and SpeI and then cloned into precut pMIR-Report luciferase vector to generate the luciferase constructs. HEK-293T cells were transfected with the indicated plasmids and 2 ng pRL-TK vector along with 100 nM of miR-29c mimics or negative control. Luminescence was measured by the dual-luciferase reporter assay system (Promega, WI, USA). Renilla luciferase activity was normalized with the Firefly luciferase and expressed as relative light units (RLU).

### Statistics

All the experiments were repeated at least three times and data were expressed as the mean ± SD. Statistical significance between two groups was analyzed by two-tailed Student's *t* test using Prism software (GraphPad 7.0). *P* < 0.05 was assumed to indicate statistical significance.

## Results

### miR-29c inhibits gastric cancer cell migration and invasion

**Figure 1:**
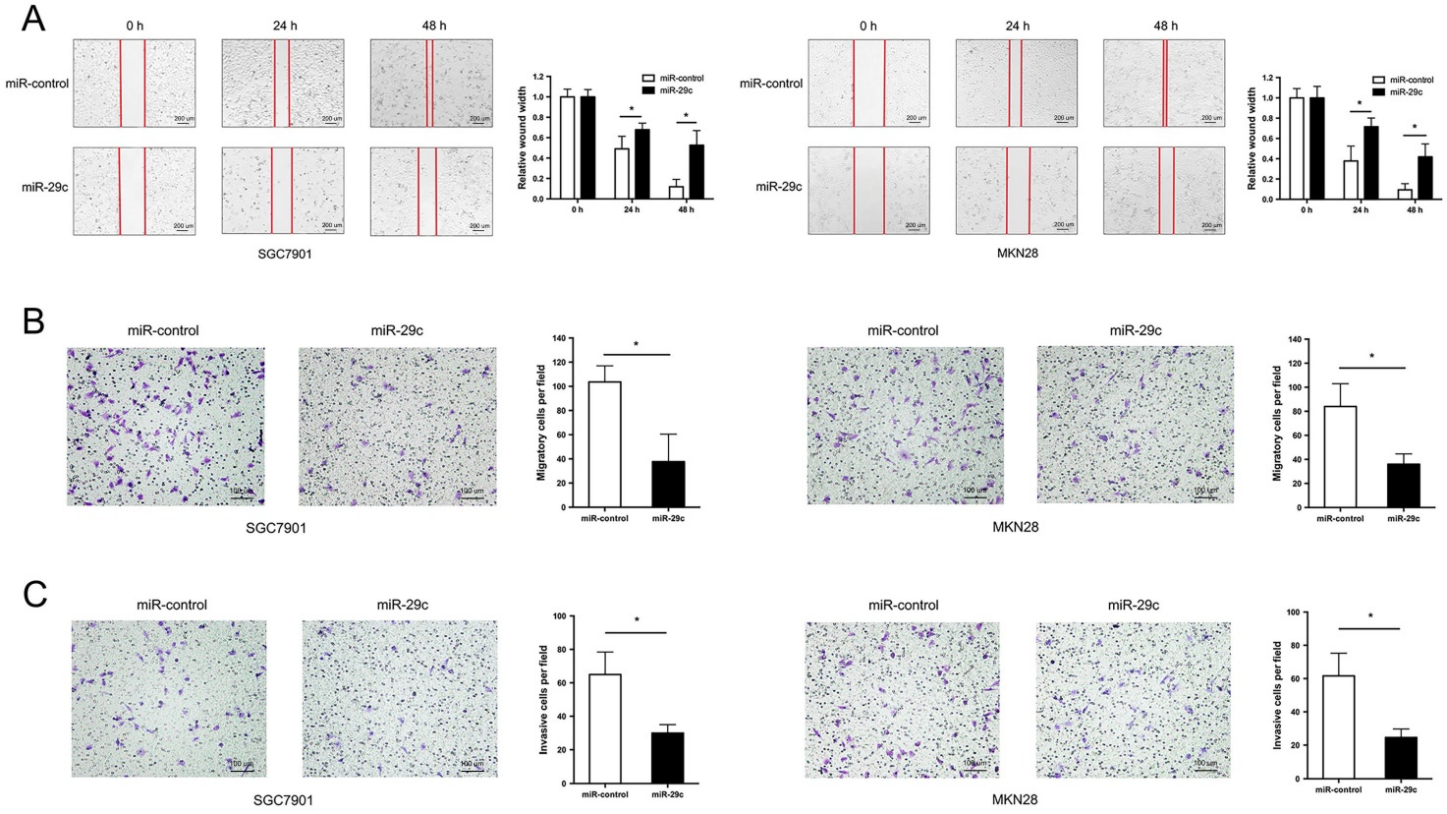
** miR-29c inhibits migration and invasion in GC cells. A.** Wound healing ability of miR-control or miR-29c from transfected SGC7901 cells (left panel) and MKN28 cells (right panel) at 24 h and 48 h. **B.** Transwell migration assay of miR-control or miR-29c transfected SGC7901 and MKN28 cells.** C.** Invasion assay of the two groups in SGC7901 and MKN28 cells. Data are represented as the mean ± SD from three independent experiments. Microscope magnification: 40×. *P < 0.05.

Two GC cell lines SGC7901 and MKN28 with the lowest expression of miR-29c were transfected with LV-miR-29c mimics to establish SGC7901-miR-29c and MKN28-miR-29c stable clones for miR-29c overexpression. Wound healing and transwell assays were performed to detect cell migration and invasion abilities. The results showed that compared with the relative control group, the width of wound closures in the miR-29c upregulated groups was significantly increased in both 24 h and 48 h (Figure 1A). Furthermore, overexpression of miR-29c resulted in a significant decrease in the number of migratory and invasive cells (Figure 1B, 1C). These data indicate that miR-29c could inhibit the migration and invasion of GC cells *in vitro*.

### miR-29c suppresses gastric cancer cell angiogenesis

**Figure 2:**
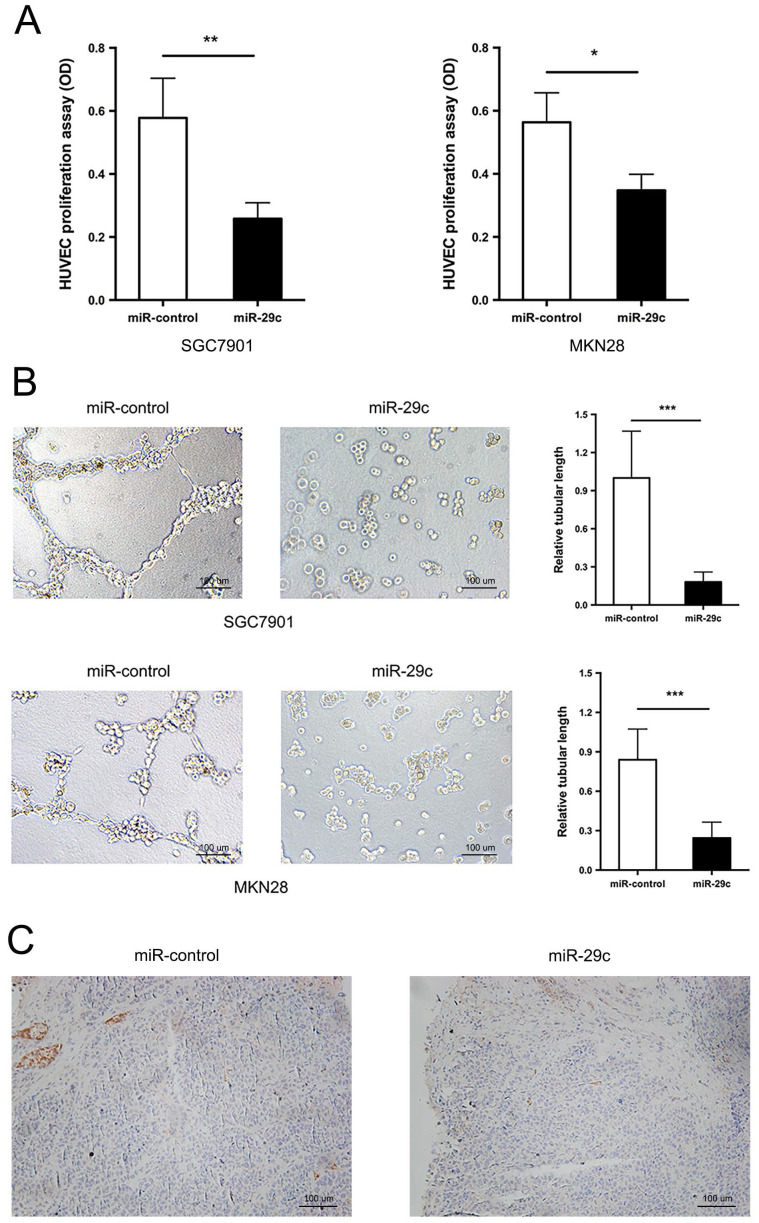
** miR-29c inhibits cell angiogenesis. A.** Proliferation of HUVECs was measured by CCK8 after 72 h in the tumor conditioned medium from miR-control or miR-29c transfected SGC7901 (left panel) and MKN28 (right panel) cells. **B.** Tube formation ability of HUVECs was assessed by tube formation assay after cultivation for 6 h in the tumor conditioned medium from miR-control or miR-29c transfected gastric cancer cells.** C.** Immunohistochemical staining of CD31 expression in mice tumor sections from SGC7901-miR-29c and control groups. These data are shown as the mean ± SD of three independent experiments. Microscope magnification: 100×. *P < 0.05; **P < 0.01; ***P < 0.001.

In addition to the strong metastatic characteristic inhibition, miR29c was observed to have lower capacity of angiogenesis. The tumor conditioned medium from SGC7901-miR-29c, MKN28-miR-29c and their relative control groups were collected and used to perform HUVECs proliferation and tube formation assays. The ability of HUVECs to proliferate in the tumor-conditioned medium from SGC7901-miR-29c and MKN28-miR-29c cells was in a significant decrease, compared with that from SGC7901-miR-control and MKN28-miR-control cells (Figure 2A). Moreover, HUVECs cultured in the tumor-conditioned medium from miR-29c overexpression cells developed fewer and smaller tubules than those that grew in the medium from control cells (Figure 2B). The expression level of CD31, specific marker of endothelial cells, was also investigated in subcutaneous xenograft model. The tumor derived from SGC7901-miR-29c showed less CD31 staining than that derived from control (Figure 2C). Therefore, these results reveal that miR-29c suppressed GC cell angiogenesis *in vitro*.

### miR-29c inhibits gastric cancer cell metastasis *in vivo*

**Figure 3:**
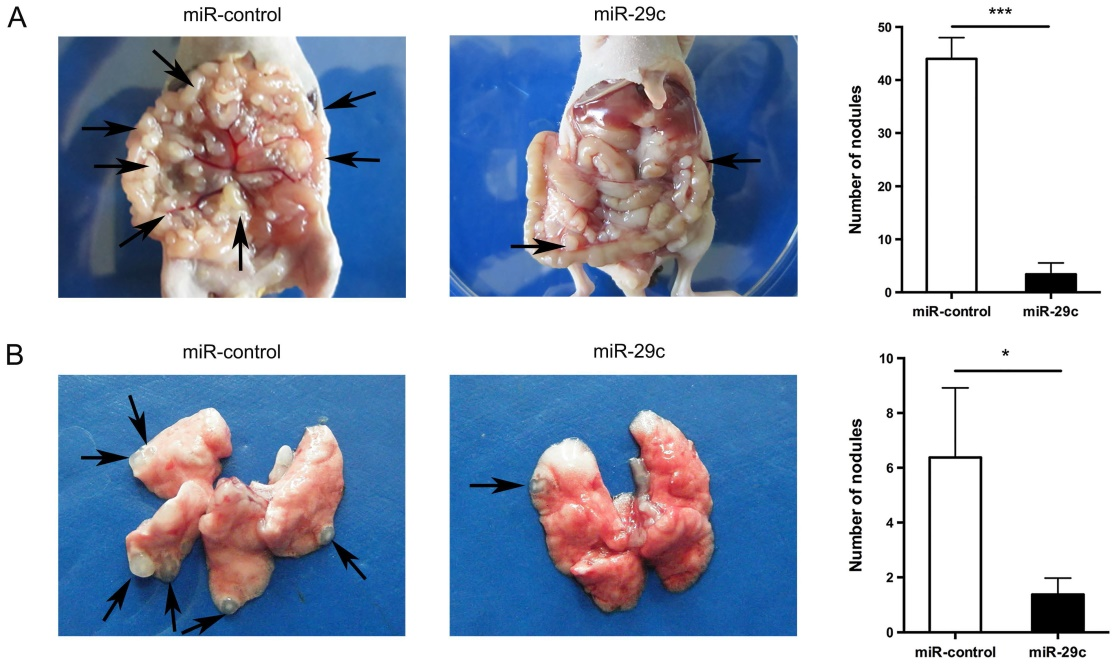
** Overexpression of miR-29c inhibits GC cell metastasis *in vivo*. A.** Representive photographs of tumors and number quantification of disseminated metastatic tumor nodules derived from SGC7901-miR-29c cells and the control cells in nude mice intraperitoneal model.** B.** Effects of miR-29c on nude mice pulmonary metastasis. The number of mice was at least 5 in each group. Arrows indicated metastatic nodules. *P < 0.05; ***P < 0.001.

To further investigate the effect of miR-29c overexpression in cell metastasis, we successfully established mice model mimicking peritoneal or lung metastasis by intraperitoneally or tail vein injecting SGC7901-miR-29c and control group cells. The nude mice of miR-29c overexpressing group were demonstrated significantly decreased peritoneal nodules and lung metastatic tumors compared with that of control group (44.00 ± 4.00 *vs.* 3.40 ± 2.14, n = 5, *P* < 0.001, Figure 3A; 5.37 ± 1.73 *vs.* 1.37 ± 0.60, n = 8, *P* = 0.04, Figure 3B). These data show that miR-29c strongly inhibits GC cell metastasis *in vivo*.

### miR-29c attenuates EMT and stemness maintenance

**Figure 4:**
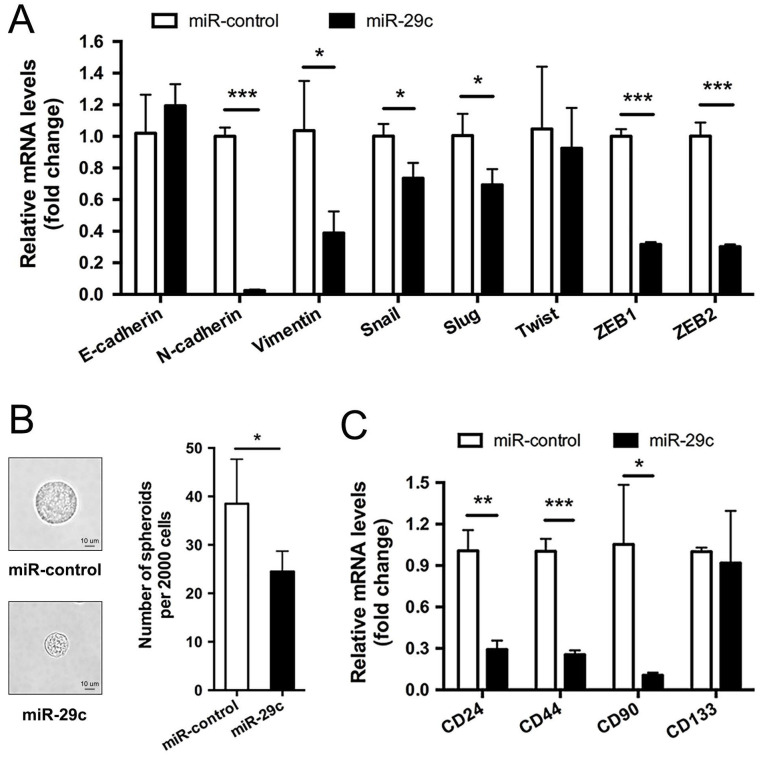
** miR-29c attenuates EMT and stemness maintenance. A.** EMT markers including E-cadherin, N-cadherin, Vimentin, Snail, Slug, Twist, ZEB1 and ZEB2 expression in SGC7901-miR-control and SGC7901-miR-29c by qRT-PCR. **B.** Representative images and statistical graph of tumor spheres from SGC7901-miR-control and SGC7901-miR-29c cells.** C.** CD24, CD44, CD90 and CD133 were detected by qRT-PCR as stemness markers between SGC7901-miR-control and SGC7901-miR-29c groups. Data are from three independent experiments. *P < 0.05; **P < 0.01; ***P < 0.001.

Typically, high capacity of cancer cells for metastasis is accompanied by functional gain of epithelial-to-mesenchymal transition (EMT) and stemness 13. Therefore, we first examined EMT markers in SGC7901-miR-29c and SGC7901-miR-control cells. The result showed that, compared with control group, the epithelial marker E-cadherin was slightly upregulated in miR-29c cells though without significant *P* value, the mesenchymal markers N-Cadherin and Vimentin were dramatically downregulated in miR-29c cells, and the transcription factors Snail, Slug, Twist, ZEB1 and ZEB2 that have been identified as EMT regulators also declined during this process (except Twist, *P* < 0.05; Figure 4A). The differential expression pattern of these EMT-related markers between the miR-29c and control groups suggests that the overexpression of miR-29c tend to attenuate the mesenchymal traits of GC cells. Furthermore, sphere formation efficiency of miR-29c cells were significantly decreased than that of control cells (Figure 4B). Moreover, the expression level of stem cell markers such as CD24, CD44, CD90, CD133 were reduced in miR-29c cells to a varying degree (Figure 4C). Together, these results suggest that miR-29c attenuates stemness properties in GC cells.

### VEGFA is the direct target of miR-29c

**Figure 5:**
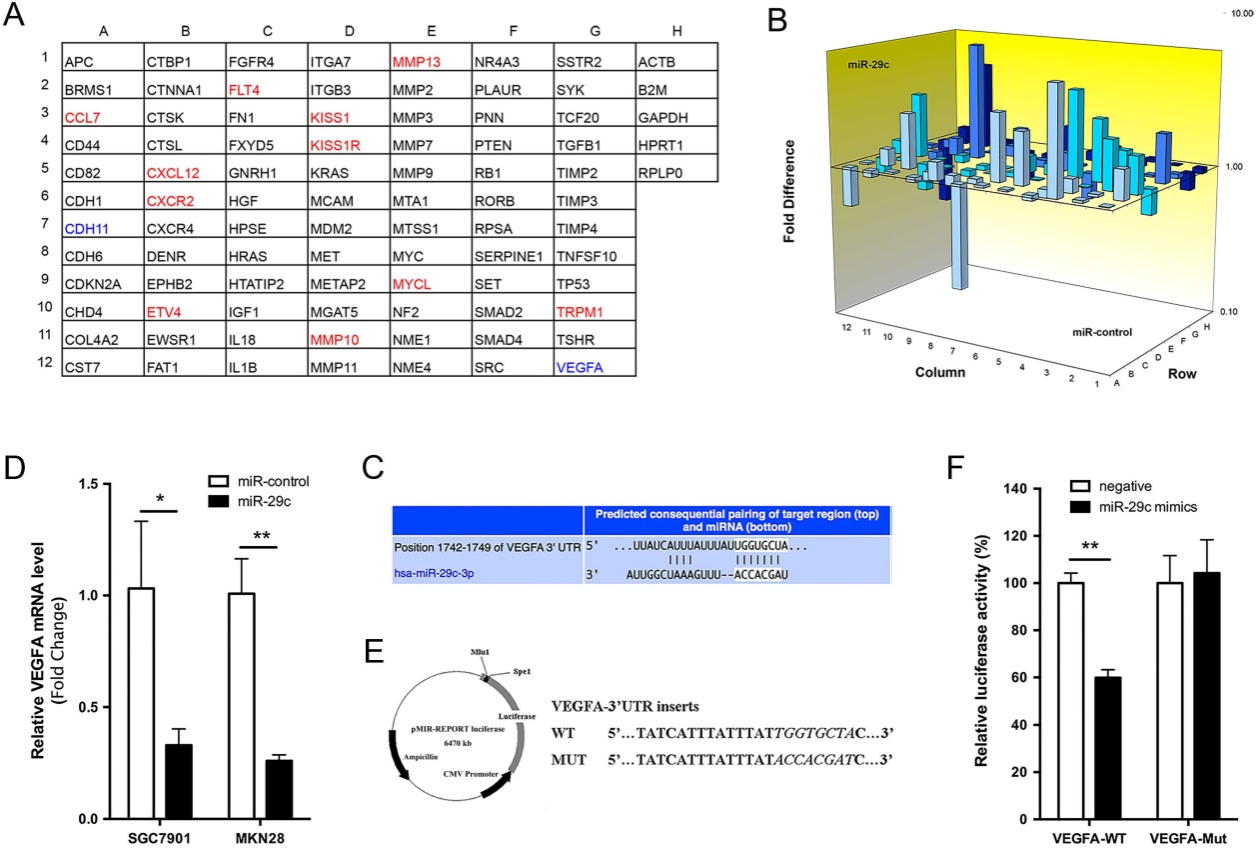
** Tumor metastasis gene profile of SGC7901-miR-29c and SGC7901-miR- control cells. A.** Grid represents array of 84 tumor metastasis-related genes (column A-G) and 5 house keeping genes (column H); red: gene expression higher in SGC7901-miR-29c cells (≥ 2.0 fold compared with SGC7901-miR-control cells); green: gene expression lower in SGC7901-miR-29c cells (≤ 2.0 fold *vs.* control cells).** B.** 3D Profile comprising 84 tumor metastasis-related genes expression in SGC7901-miR-29c and SGC7901-miR-control cells.** C.** Schematic representation of a putative binding site of miR-29c in the VEGFA-3'UTR predicted by TargetScan.** D.** QRT-PCR validated the differences of VEGFA expression level between miR-29c and miR-control groups both of SGC7901 and MKN28 cells.** E.** Schematic illustration of the reporter plasmids. **F.** Dual-luciferase assay was performed on HEK-293T cells. Means ± SD are shown. *P < 0.05; **P < 0.01.

To explore the molecular mechanisms through which miR-29c exerts its function in GC metastasis, we performed a tumor metastasis PCR array that is comprised of 84 metastasis-related genes on SGC7901-miR-29c and SGC7901-control cells. Eleven genes were increased more than two-fold by miR-29c overexpression, while only CDH11 and VEGFA were reduced more than half (Figure 5A, 5B). Using the TargetScan database, we found that a separate miR-29c-binding seed sequence was conserved through evolution in 3'UTR of VEGFA gene (Figure 5C). Overexpression of miR-29c decreased the mRNA expression of VEGFA, both upon in SGC7901 and MKN28 cells (Figure 5D). To demonstrate if there was a direct interaction between miR-29c and VEGFA, the pMIR-REPORT luciferase reporters containing a miR-29c binding site (VEGFA-3'UTR-WT) or a mutated site (VEGFA-3'UTR-MUT) were constructed (Figure 5E). These vectors were co-transfected with miR-29c mimics or negative controls into 293T cells. Dual-luciferase reporter assay showed that the luciferase activity in miR-29c group was decreased by 40%, compared to negative controls (*P* < 0.01, Figure 5F). Meanwhile, miR-29c mimics did not affect the luciferase activity of VEGFA-3'UTR-MUT vector. These results support that the VEGFA could be a target of miR-29c.

### VEGFA overexpression restores the miR-29c induced inhibition of cell metastasis

**Figure 6:**
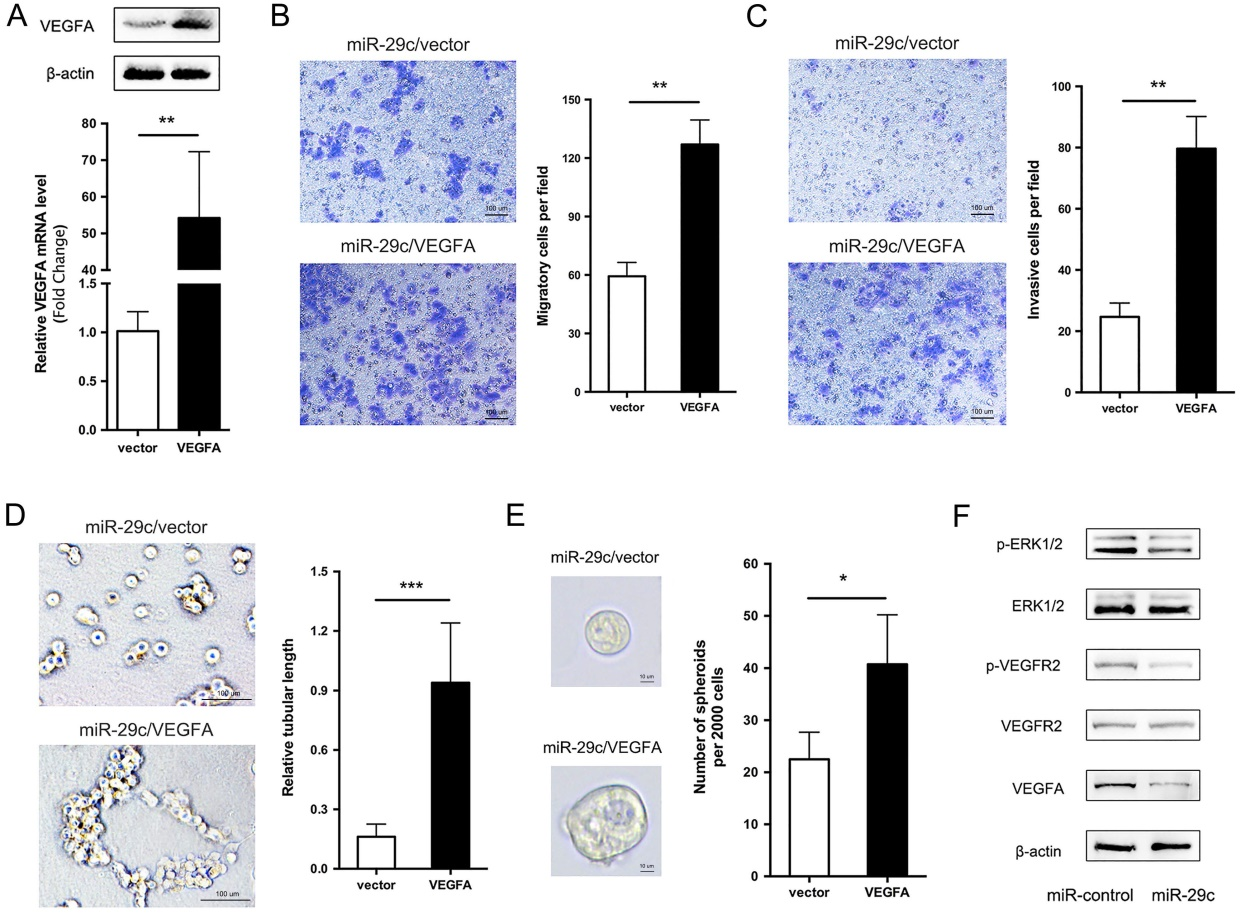
** VEGFA overexpression elicits the phenotypes of miR-29c in SGC7901 cells. A.** VEGFA overexpression efficiency was evaluated in SGC7901-miR29c cells transfected with VEGFA or empty vector by qRT-PCR and Western blot analysis.** B.** Representative images and statistical graph of migration assay between SGC7901-miR29c/VEGFA and vector cells.** C.** Invasion assay in the two groups, representative images and statistical graph was shown.** D.** Representive photographs of tube formation ability between the two groups.** E.** Sphere formation in SGC7901-miR29c/VEGFA and SGC7901-miR29c vector cells.** F.** Western blot analysis of VEGFA/VEGFR2/ERK pathway in SGC7901-miR-control and SGC7901-miR-29c cells. Means ± SD are shown. *P < 0.05; **P < 0.01; ***P < 0.001.

We showed that miR-29c stable expression remarkably suppressed cell migration, invasion, tubular formation and sphere formation, and decreased the expression of VEGFA. Given that VEGFA is a direct target of miR-29c, then, we investigated whether VEGFA decrease is responsible for miR-29c induced GC cell metastasis inhibition. To this end, VEGFA overexpression constructs were introduced into SGC7901-miR-29c cells. VEGFA mRNA and protein level was verified by qRT-PCR and Western bolt in Figure 6A. Compared to vector, VEGFA ectopic expression in SGC7901-miR-29c cells significantly improved the cell migrate and invasive capabilities (Figure 6B, 6C). In addition, because the SGC7901-miR-29c cells showed the ability lost of tubular formation compared to SGC7901-control cells, expression of VEGFA in SGC7901-miR-29c cells led to the recovery of tubular formation (Figure 6D). Moreover, sphere formation ability was also recovered after VEGFA overexpression in SGC7901-miR-29c cells (Figure 6E). Taken together, these data indicate that the overexpression of VEGFA could restore the suppressed metastatic capacities induced by miR-29c in GC cells.

### miR-29c inhibits VEGFA/VEGFR2/ERK pathway to suppress GC cell metastasis

It has previously been shown that VEGFA usually combines with its receptor VEGFR2 to promote cell migration, invasion and angiogenesis 14. Thus, we observed the related protein levels in SGC7901-miR-29c and control cells. The total protein level of VEGFR2 had no change between two groups. However, the phosphorylation level of VEGFR2 in SGC7901-miR-29c cells showed an obvious decline compared with that in SGC7901-miR-control cells. The phosphorylation of VEGFR2 has been reported that can further activate extracellular signal-regulated kinase (ERK) signaling. And ERK pathway is involved in regulation of cell proliferation, differentiation, motility, angiogenesis and survival. Therefore, we detected the total and the phosphorylation levels of ERK1/2 in GC cells transfected with miR-29c and found that the phosphorylate ERK1/2 was decreased compared with the control cells within the stable level of total ERK1/2 (Figure 6F). This reveals that VEGFA/VEGFR2/ERK pathway plays a role in this regulatory process through which miR-29c downregulates VEGFA expression and inhibits metastasis ability in GC cells.

## Discussion

Metastasis is one of the major contributors to deaths from cancer worldwide. EMT and CSCs existence are perceived to be responsible for cancer metastasis 13. The process of EMT has been linked to CSCs property acquisition and CSCs manifest EMT traits meanwhile, further highlights the close association between the properties of CSCs and EMT during metastasis 15-17. Presently, it remains largely unclear whether a master regulator or a common pathway exists to promote EMT and CSCs maintenance. Advances in this field will enable the development of novel and efficient GC therapy approaches.

Abundant miRNAs play essential roles in a variety of biological processes, such as cell proliferation, migration, invasion, angiogenesis and cell differentiation 18. Growing evidence has demonstrated that dysregulation of miRNAs is associated with metastatic processes in various kinds of cancers and can function as oncogenes or tumor suppressors 19, 20. The miR-29 family members, including miR-29a, miR-29b and miR-29c, function as tumor suppressors and are generally downregulated in human cancers, such as breast cancer, colorectal cancer, non-small cell lung cancer and aggressive chronic lymphocytic leukemia 21-24. Our previous research found that miR-29c was downregulated in GC tissues and overexpressed miR-29c inhibited cell proliferation, promoted cell apoptosis and arrested cell cycle at G1/G0 phase 9. Compared with the previous article that mainly studied its role in proliferation, in this study we focused on its role in metastasis. Overexpressing miR-29c not only inhibited the abilities of cell migration, invasion and angiogenesis, but also attenuated GC cell EMT and stemness properties *in vitro* and *in vivo*.

**Figure 7:**
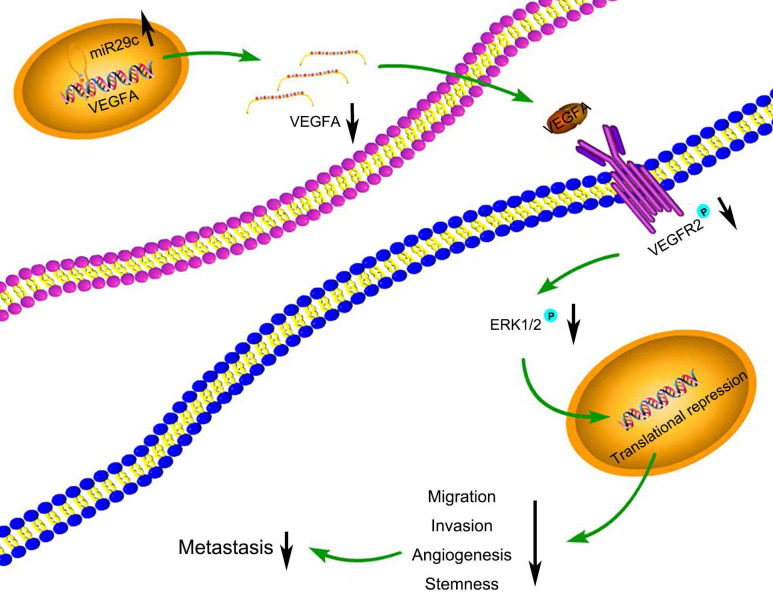
** Schematic diagram summarizing the interplay among miR-29c-VEGFA/VEGFR2/ERK pathway through which miR-29c inhibits GC cell metastasis.** miR-29c functions as a metastasis suppressor by targeting VEGFA to inhibit GC cell metastasis through the modulation of VEGFA/VEGFR2/ERK pathway.

Many target mRNAs of miR-29c, including FOXP1, CDK6, NASP and CTNND1 in GC, have been discovered 9, 25-27. The modulation of miR-29c in our study revealed a crucial role in the regulation of VEGFA levels, suggesting that it is a molecular regulator of metastatic function via miR-29c/VEGFA signaling. By contrast, overexpression of VEGFA significantly increased metastatic abilities in GC cells transfected with miR-29c. Moreover, in this study, miR-29c decreased the ERK1/2 phosphorylation in GC cells, which revealed that ERK signaling was participated in the miR-29c-mediated regulation of VEGFA. It has been well recognized that VEGFA combines with VEGFR2, phosphorylates and activates VEGFR2, which further activates ERK signaling 14, 28. In our study, miR-29c inhibited the phosphorylation of VEGFR2 and ERK1/2 through VEGFA downregulation in GC cells. Herein, this VEGFA/VEGFR2/ERK signaling might be a critical mechanism by which miR-29c inhibits GC metastasis (Figure 7). Elucidating the mechanisms and pathways involved in invasive phenotypes increases our understanding of cancer metastasis and helps to identify novel molecular targets to eradicate GC.

Currently, anti-tumor drugs have been demonstrated to target VEGF signaling, and among them, Bevacizumab has been in wide use for treatment of multiple cancers, including GC 29, 30. However, the efficiency of this drug on GC patients' needs to be further improved. As an upstream mediator of VEGFA, miR-29c could inhibit GC cell angiogenesis and metastasis and thus might be a promising therapeutic intervention to enhance treatment efficacy and postpone the progression of GC.

Great efforts have been made to elucidate the molecular mechanisms underlying the aggressive behavior of GC and search for effectors involved in diverse biological processes related to metastasis. In the present study, we identified a significant role for miR-29c in regulating migration, invasion and angiogenic properties of GC cells. This function of miR-29c is mediated by VEGFA at the post-transcriptional level. The results of our study suggest that miR-29c-VEGFA/VEGFR2/ERK pathway may act as potential anti-metastatic targets for treatment of GC.

## Conclusion

Summarizing our findings here, this study elucidates that miR-29c is an inhibitor for cell migration, invasion and angiogenesis through regulation of EMT and stemness properties in GC. Furthermore, we illuminate that miR-29c regulates metastasis through effecting the promoter activity of VEGFA and cooperates with ERK1/2 to supervise GC metastasis progression. Therefore, targeting components of the miR-29c-VEGFA/VEGFR2/ERK axis may provide novel therapeutic strategies for the prevention of GC metastasis.

## Supplementary Material

Supplementary figure and table.

## Abbreviations

GC: gastric cancer
VEGF: vascular endothelial growth factor
EMT: epithelial-mesenchymal transition
CSCs: cancer stem cells
HUVECs: human umbilical vein endothelial cells
HEK-293T: human embryonic kidney 293T cells
ERK: extracellular signal-regulated kinase
