# Supplementary material for: miR-29c inhibits metastasis of gastric cancer cells by targeting VEGFA
Source: J Cancer. 2022 Oct 31;13(14):3566–74. doi: 10.7150/jca.77727 (PMC9723994; doi:10.7150/jca.77727)
Supplement: Supplementary file 1 — Supplementary figure and table. [file jcav13p3566s1.pdf]

Supplementary Materials

Table S1. Primers used in this study

Primer Name	Sequence (5' -> 3')
E-cadherin Forward	CGAGAGCTACACGTTCACGG
E-cadherin Reverse	GGGTGTCGAGGGAAAAATAGG
N-cadherin Forward	AGCCAACCTTAACTGAGGAGT
N-cadherin Reverse	GGCAAGTTGATTGGAGGGATG
Vimentin Forward	GACGCCATCAACACCGAGTT
Vimentin Reverse	CTTTGTCGTTGGTTAGCTGGT
Snail Forward	TCGGAAGCCTAACTACAGCGA
Snail Reverse	AGATGAGCATTGGCAGCGAG
Slug Forward	TGTGACAAGGAATATGTGAGCC
Slug Reverse	TGAGCCCTCAGATTTGACCTG
Twist Forward	GTCCGCAGTCTTACGAGGAG
Twist Reverse	GCTTGAGGGTCTGAATCTTGCT
ZEB1 Forward	CAGCTTGATACCTGTGAATGGG
ZEB1 Reverse	TATCTGTGGTCGTGTGGGACT
ZEB2 Forward	CAAGAGGCGCAAACAAGCC
ZEB2 Reverse	GGTTGGCAATACCGTCATCC
CD24 Forward	CTCCTACCCACGCAGATTTATTC
CD24 Reverse	AGAGTGAGACCACGAAGAGAC
CD44 Forward	CTGCCGCTTTGCAGGTGTA
CD44 Reverse	CATTGTGGGCAAGGTGCTATT
CD90 Forward	ATCGCTCTCCTGCTAACAGTC
CD90 Reverse	CTCGTACTGGATGGGTGAACT
CD133 Forward	AGTCGGAAACTGGCAGATAGC
CD133 Reverse	GGTAGTGTTGTACTGGGCCAAT
VEGFA Forward	AGGGCAGAATCATCACGAAGT
VEGFA Reverse	AGGGTCTCGATTGGATGGCA
GAPDH Forward	TTGGCATCGTTGAGGGTCT
GAPDH Reverse	CAGTGGGAACACGGAAAGC

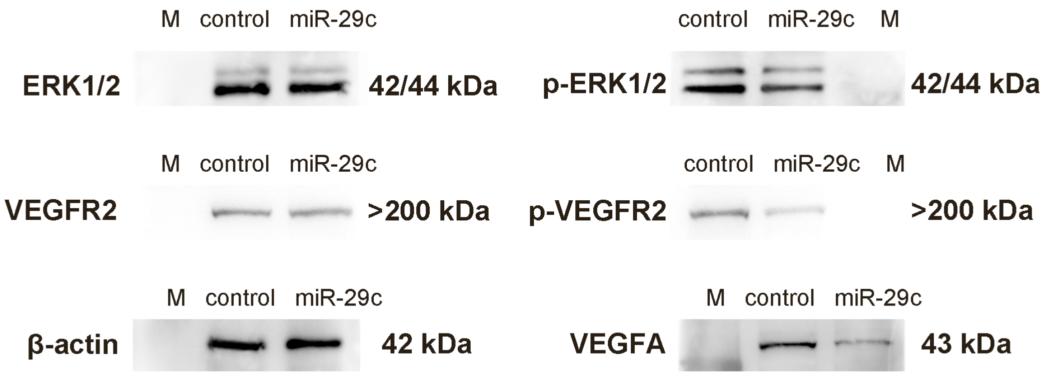

Figure S1. Original uncropped Western blots shown in main text (related to Figure 6F).
